# Reliability and validity of psychosocial and environmental correlates measures of physical activity and screen-based behaviors among Chinese children in Hong Kong

**DOI:** 10.1186/1479-5868-8-16

**Published:** 2011-03-08

**Authors:** Ya-Jun Huang, Stephen H Wong, Jo Salmon, Stanley S Hui

**Affiliations:** 1 Department of Sports Science and Physical Education, The Chinese University of Hong Kong 00852, PR China; 2 Centre for Physical Activity and Nutrition Research, School of Exercise and Nutrition Sciences, Deakin University, Burwood 3125, Victoria Australia

## Abstract

**Background:**

Insufficient participation in physical activity and excessive screen time have been observed among Chinese children. The role of social and environmental factors in shaping physical activity and sedentary behaviors among Chinese children is under-investigated. The purpose of the present study was to assess the reliability and validity of a questionnaire to measure child- and parent-reported psychosocial and environmental correlates of physical activity and screen-based behaviors among Chinese children in Hong Kong.

**Methods:**

A total of 303 schoolchildren aged 9-14 years and their parents volunteered to participate in this study and 160 of them completed the questionnaire twice within an interval of 10 days. Intraclass correlation coefficients (ICCs), kappa statistics, and percent agreement were performed to evaluate test-retest reliability of the continuous and categorical variables, respectively. Exploratory factor analyses (EFAs) were conducted to assess convergent validity of the emergent scales. Cronbach's alpha and ICCs were performed to assess internal and test-retest reliability of the emergent scales. Criterion validity was assessed by correlating psychosocial and environmental measures with self-reported physical activity and screen-based behaviors, measured by a validated questionnaire.

**Results:**

Reliability statistics for both child- and parent-reported continuous variables showed acceptable consistency for all of the ICC values greater than 0.70. Kappa statistics showed fair to perfect test-retest reliability for the categorical items. Adequate internal consistency and test-retest reliability were observed in most of the emergent scales. Criterion validity assessed by correlating psychosocial and environmental measures with child-reported physical activity found associations with physical activity in the self-efficacy scale (*r *= 0.25, *P *< 0.05), the peer support for physical activity scale (*r *= 0.25, *P *< 0.05) and home physical activity environmental (*r *= 0.14, *P *< 0.05). Children's screen-based behaviors were associated with the family support for physical activity scale (*r *= -0.22, *P *< 0.05) and parental role modeling of TV (*r *= 0.12, *P *= 0.053).

**Conclusions:**

The findings provide psychometric support for using this questionnaire for examining psychosocial and environmental correlates of physical activity and screen-based behaviors among Chinese children in Hong Kong. Further research is needed to develop more robust measures based on the current questionnaire, especially for peer influence on physical activity and parental rules on screen-based behaviors.

## Background

The beneficial effects of physical activity on both physical (e.g. prevention of obesity) and mental health problems have been well established in children [1]. Although there is a paucity of data on the associations of physical activity and chronic disease risk factors in Chinese children, a recent citywide survey in Hong Kong found that children and adolescents with higher physical activity had lower cardiovascular risk scores [2]. Further, this association was independent of age and puberty stage. Despite this, physical activity levels of Hong Kong children seem to be not enough to achieve health benefits. In the abovementioned citywide survey, only 21.5% of the respondents reported a high level of physical activity assessed by a physical activity rating scale [2]. The sedentary lifestyle of Hong Kong children has been also reported in another study [3], which showed that they spent nearly twice the time on electronic media use than on health-enhancing physical activity. In order to develop effective strategies to promote a more active lifestyle, understanding the factors that influence children's physical activity and sedentary behaviors is essential. Recently, research focus has centred on determining how the social and physical environment might relate to health-related behaviors among youth [4-6]. The importance of the ecological niche has been emphasized to better understand development or change in individual behavior [7].

Using the social ecological framework, it has been identified in a recent review that certain individual (e.g. self-efficacy), social (e.g. parents and family influences, friend support), and environmental factors (e.g. access to recreational facilities) have an important impact on children's physical activity [6]. However, conclusions regarding the correlates of sedentary behaviors can not be drawn due to the insufficient evidence in children [6] and preschoolers [8]. Furthermore, recent reviews [4,6,9,10] have highlighted that most studies have relied on either self-reported [11-16] or parent-reported [13,15-17] perceptions of the social and physical environments to establish the relationships between correlates and physical activity.

Several studies have reported the psychometric properties of instruments assessing psychosocial correlates [18-20] or certain aspects of the physical environment in the home and neighborhood [11,12,15,21] and physical activity or sedentary behavior in diverse youth populations. In The Trial of Activity in Adolescent Girls (TAAG), a multicenter study, a survey instrument was developed to assess girls' perceptions of physical environment in relation to physical activity. Kappa statistics indicated fair to moderate test-retest reliability among 480 sixth- and eighth-grade girls in US communities [12]. A broader range of factors were considered in assessing the physical environment in the home and the neighborhood among Australian children [21]. The results suggested that the instrument was reliable in most items with kappa values higher than 0.80. In another study among 9- to 12-year-old children in the US, an instrument designed to assess perceived environmental access to physical activity at home, school, and in the neighborhood [22] was found to be generally reliable.

Although there is some evidence of psychometric properties of psychosocial and environmental correlates measures of physical activity in youth, most of the studies have been conducted among Caucasian children and adolescents. Little is known regarding whether the correlates that have been extensively examined in previous studies could be applied in under-represented populations such as Chinese children in Hong Kong. Moreover, few studies have considered the social and environmental correlates of sedentary behaviors, which are primarily screen-based behaviors such as watching television (TV), using the computer and playing electronic games [15,23]. Therefore, the purpose of this study was to examine the reliability and validity of a questionnaire to measure child- and parent-reported psychosocial and environmental correlates of physical activity and screen-based behaviors among Chinese children in Hong Kong.

## Methods

### Participants

A sample of 303 children (143 boys and 160 girls) aged 9-14 years were recruited from 7 primary schools through written descriptions distributed to parents by school teachers. To ensure the sample provided a reasonable representation of families from different socio-economic status (SES) areas, the schools were located in five different districts in Hong Kong of varying SES, i.e. one with high SES, one with low SES and the other three with medium SES, according to local statistics [24]. Consent forms were sought from parents and the participating children. The study was approved by the University Survey and Behavioral Research Ethics Committee.

### Questionnaire development

The present study was conducted from March to June 2007 as a part of a study survey aimed at investigating the correlates of physical activity and sedentary behaviors of Hong Kong Chinese children. The social ecological model [7] was used to develop the questionnaire for Chinese children and parents in assessing the psychosocial and environmental correlates of the children's physical activity and screen-based behaviors. Although this model has been extensively applied in developing different measures related to physical activity in other populations, these measures have not been assessed in Hong Kong children. The variables in the questionnaires were chosen based on the most promising data from recent reviews [6], feasibility and policy relevance. Most of the individual items in the questionnaire were adapted from previously used questions while a few of them were newly established. Child-reported questions derived from previous studies included: self-efficacy [25], family and peer support for physical activity [21], perceived parental enjoyment of screen-based behaviors together [15], perceived neighborhood safety [21], social environment in the neighborhood [21] and sports facilities in the neighborhood [11,21]. Parent-reported items adapted from previously used instruments covered: rules and guidance on children's screen-based behaviors [15] and sedentary opportunities in the home [21]. Child-reported home physical activity environment and parent-reported role modeling for physical activity and screen-based behaviors were newly developed questions. Although a similar construct has been developed in previous studies regarding physical activity environment in the home, with limited home space and high population density it is less likely that families of Hong Kong children own a swimming pool, front fence, or other outdoor areas at home, which were measured in previous studies [13,21].

The questions derived from previous studies were translated into Chinese by two investigators who are both Chinese native speakers but also speak English. In addition to abovementioned psychosocial and environmental measures, demographic information was also collected in the questionnaire regarding responding parents' age, sex, education level, occupation and marital status (married, widowed, divorced/separated and unmarried) as well as the children's date of birth, sex and number of siblings in the home. A pilot test was conducted in a small group of 18 children of a similar age and their parents to ensure the suitability and feasibility of the questions. Few amendments were made following the feedback from the children and parents. A description of the psychosocial and environmental measures in the questionnaire is provided in Additional File 1.

The children were surveyed during physical education (PE) classes or school recess during school visits with the investigators present. Meanwhile, the parent of each participating child completed part of the questionnaire at home. They were instructed to return the forms to the teachers in one to two weeks. The questionnaire was administered on two occasions, approximately 10 days apart, to assess the test-retest reliability. It was specified to the participants that the responding parent should be the same for the two administrations.

### Children's physical activity and screen-based behaviors

Physical activity was assessed using the Children's Leisure Activities Study Survey questionnaire-Chinese version (CLASS-C) which has been validated for Chinese children in Hong Kong [26]. The questionnaire included a checklist of 31 physical activities and screen-based behaviors, i.e., watching TV, using the computer, and playing electronic games. Children reported the total time they spent in these activities during weekdays and weekend days respectively in the past week. The intensity of physical activity was determined according to a published compendium [27]. The questionnaire was then scored by calculating the daily time spent in moderate-to-vigorous physical activity (MVPA) and screen-based behaviors.

### Data analysis

Statistical analyses were performed using SPSS for Windows version 17.0. Exploratory factor analyses (EFAs) were conducted to determine the convergent validity on the self-efficacy scale, the family and peer support for physical activity scale, the perceived neighborhood safety scale, the social environment in the neighborhood scale, and the rules and guidance on children's screen-based behaviors scale, using data from the first administration. As the data were not normally distributed, principal axis factoring was used. If more than one factor was generated, direct oblimin rotation was conducted to determine the factor patterns. For each analysis, factors with an eigenvalue less than 1.00 were dropped. Factor loadings equal or greater than 0.30 were considered to be significant. Kaiser-Meyer-Oklin value (>0.60) and Bartlett's Test of Sphericity (*P *< 0.05) were explored to determine the factorability of the data. Also, any individual variables with high percentage of variance unexplained by the factors that emerged were removed from the analysis, as this suggests that the variable cannot be explained well by the common factors identified [28]. Once the factors were determined, items within each identified scale were averaged (for continuous items) or summed (for dichotomous items), and then Cronbach's alpha coefficients were calculated. Internal reliability was deemed acceptable if the Cronbach's α was greater than 0.70 [21]. Intraclass coefficient correlations (ICCs) were calculated to assess the test-retest reliability of the scales and those individual continuous items comprising the scales. Adequate reliability was reached when the ICC values were greater than 0.70 [29]. Kappa statistics were used to examine the test-retest reliability of the dichotomous variables. The relative strength of agreement was interpreted according to the classification scales for kappa [30]: 0.00-0.20 poor, 0.21-0.40 fair, 0.41-0.60 moderate, 0.61-0.80 substantial, and 0.81-1.00 almost perfect. Since the stability of kappa values is influenced by the prevalence of responses [31], percent agreement was also calculated. Percent agreement values greater than 66% were classified as fair [32]. Criterion validity of the psychosocial and environmental measures was determined by computing Pearson correlations between these variables with self-reported physical activity and screen-based behaviors.

## Results

The descriptive statistics for the demographic characteristics are shown in Table 1. All the participants were Chinese and from grades 4 to 6; 72.7% had at least one sibling. Among the responding parents, approximately 71% were mothers. The mean age of the responding parents was about 42 years old. Approximately 50% of the mothers and 8% of the fathers were unemployed. More than 90% of the responding parents were married. Nineteen of the mothers or fathers attained tertiary education. The participating children and responding parents did not report any difficulties in understanding the survey questions. The children self-reported participation in an average of 74 minutes of MVPA per day and more than two hours of screen time. Approximately 47% of the children did not reach the physical activity recommendation of 60 minutes daily [33]. No sex differences were observed in MVPA and screen time. For five of the total 160 children who completed the questionnaire twice in order to determine the test-retest reliability, the responding parents were found to be different for the two administrations. Therefore, the data from these parents were excluded in the reliability analysis of parent-report questions. The missing rates were approximately 3% and 8% for children-reported and parent-reported items, respectively.

**Table 1:** Social and general characteristics of the participants

	Boys (n = 143)	Girls (n = 160)	All (n = 303)
Age (yr) (mean ± SD)	11.0 ± 0.8	11.1 ± 0.9	11.1 ± 0.9
Siblings (%)			
0	30.0	25.0	27.3
1	54.3	56.3	55.3
≥2	15.7	18.7	17.4
Responding parent (% mother)	76.0	65.4	71.1
Age of responding parent (yr) (mean ± SD)	42.9 ± 5.4	41.6 ± 6.0	42.2 ± 5.7
Employment (% no employment)			
Fathers	6.0	10.6	8.4
Mothers	48.4	50.7	49.6
Parental education* (%)			
Lower secondary or less	40.4	41.6	40.9
Completed secondary	40.3	39.3	39.8
Tertiary	19.3	19.1	19.3
Marital Status (%)			
Married	92.1	91.1	91.5
Widowed	0.8	2.1	1.5
Divorced/Separated	5.6	6.8	6.3
Unmarried	1.6	0.0	0.7
MVPA (mins/d) (mean ± SD)	72.1 ± 50.8	75.9 ± 52.7	74.1 ± 51.7
Screen time (mins/d) (mean ± SD)	135.1 ± 117.2	136.5 ± 125.5	135.9 ± 121.4

*The highest education level for either the father or the mother

MVPA: moderate-to-vigorous physical activity

The test-retest reliability for all the individual items in the questionnaire is shown in Table 2. For the continuous variables (self-efficacy, perceived parental enjoyment of screen-based behaviors together and rules and guidance on children's screen-based behaviors), the ICC vales ranged from 0.70-0.86, indicating the acceptable repeatability. Most of the categorical items showed moderate to almost perfect reliability according to kappa values (range 0.45-1.00). The items with fair kappa values included one item in the home physical activity environment measure (κ = 0.32), one item in the family and peer support for physical activity measure (κ = 0.27) and parental role modeling of computer use (κ = 0.39). However, percent agreement for these three items showed acceptable agreement level, as they were greater than 70%.

**Table 2:** Test-retest reliability of child-reported^a ^and parental-reported^b ^measures

	ICC	95%CI	Items with low ICC
Self-efficacy^a^	0.70-0.79	0.59-0.85	None
Perceived parental enjoyment of screen-based behaviors together^a^	0.72-0.77	0.63-0.85	None
Rules and guidance on children's screen-based behaviors^b^	0.72-0.86	0.60-0.90	None
	**kappa**	**Percent agreement**	**Items with fair kappa (0.21-0.40)**
Home PA environment^a^	0.32-0.52	82-92	Have sports clothes in the home
Family and peer support for PA^a^	0.27-0.76	79-88	Be physically active with grandparents
Parental role modeling^b^	0.39-0.53	73-77	Time in using computer
Sedentary opportunities in the home^b^	0.67-1.00	91-100	None
Perceived neighborhood safety^a^	0.45-0.76	85-94	None
Social environment in the neighborhood^a^	0.55-0.68	83-86	None
Sports facilities in the neighborhood^a^	0.58-0.70	83-93	None

PA: physical activity

Table 3 shows the results of the EFAs, the Cronbach's alpha and test-retest ICC values for the emergent scales. One item "*I am confident that I can walk to school instead of having to wait for a ride*" did not fit with the other self-efficacy questions and should be analyzed as an individual variable. The remaining items all loaded onto one factor which had substantial internal consistency (Cronbach's α = 0.75) and good test-retest reliability (ICC = 0.82). For the family and peer support for physical activity measures, two factors were identified, i.e. family support and peer support. However, two of the items, "*I am usually physically active with my grandparents*," and "*I usually receive encouragement from a family member to do physical activity*" had low loading on both factors. Thus, these two items were excluded from the scales. Adequate test-retest reliability was found for both scales. The internal consistency was substantial for family support (Cronbach's α = 0.74), but poor for peer support (Cronbach's α = 0.50).

**Table 3:** Factor structure and test-retest reliability of the emergent scales in the questionnaire

Scales		% Variance explained	Eigen value	Cronbach's alpha	Test-retest ICC
**Self-efficacy**		47.0	2.35	0.75	0.82
*Variable removed: Walk to school instead of having to wait for a ride*					
**Family and peer support for PA**					
Factor 1: Family support	Being physically active with whole family; with father; with mother; with sibling	28.2	2.26	0.74	0.86
Factor 2: Peer support	Being physically active with friends; receive encouragement from friends	15.3	1.22	0.50	0.78
*Variable removed: Being physical active with grandparents; receive encouragement from family member*					
**Rules and guidance on children's screen-based behaviors**					
Factor1: Guidance	Guidance during TV, computer games and Internet use	44.0	2.64	0.86	0.83
Factor 2: Rules	Control time in TV, computer & Internet use; no TV during meal time; not allow TV until homework done	20.6	1.23	0.65	0.81
**Perceived neighborhood safety**		45.5	2.25	0.71	0.89
**Social environment in the neighborhood**		55.8	2.23	0.73	0.84

PA: physical activity; TV: television

Principal axis factoring with direct oblimin rotation (if two factors are identified). Kaiser-Meyer-Olkin values range from 0.650 (social environment in the neighborhood) to 0.728 (rules and guidance). Bartlett's Test of Sphericity for all the scales are significant at *P *< 0.01.

Variables that have factor loadings < 0.30 are removed.

Two sub-scales were yielded for parental guidance and rules on children's screen-based behaviors measures. These factors accounted for 44.0% and 20.6% respectively of the total variability in the items. Adequate test-retest reliability was observed for both scales. The internal consistency was fair for the rules scale (Cronbach's α = 0.65) and substantial for the guidance scale (Cronbach's α = 0.86). One factor was identified for the perceived neighborhood safety scale. This factor accounted for 45.5% of the total variability in the items. The internal consistency for this scale was acceptable (Cronbach's α = 0.71) and test-retest reliability was adequate (ICC = 0.89). For the social environment in the neighborhood scale, one factor was also yielded which accounted for 55.8% of the total variability in the items. The scale showed acceptable internal consistency (Cronbach's α = 0.73) and test-retest reliability (ICC = 0.84). The details on factor loading, communality coefficients and Kaiser-Meyer-Olkin values for the above scales are provided in Additional File 2.

The significant correlations of the psychosocial and environmental scales with MVPA and screen time are shown in Table 4. Self-efficacy (*r *= 0.25, *P *< 0.05), home physical activity environment (*r *= 0.14, *P *< 0.05) and peer support (*r *= 0.25, *P *< 0.05) were positively associated with child-reported MVPA. There was a negative correlation between family support for physical activity and screen time (*r *= -0.22, *P *< 0.05). Parental role modeling of TV was marginally correlated with screen time (*r *= 0.12, *P *= 0.053).

**Table 4:** Correlations between child self-reported^a ^and parent-reported^b ^psychosocial, environmental scales and physical activity and screen-based behaviors (only significant values are shown)

	MVPA	Screen time
Self-efficacy^a^	0.25*	
Home PA environment^a^	0.14*	
Peer support for PA^a^	0.25*	
Family support for PA^a^		-0.22*
Parental role modeling of TV^b^		0.12^#^

PA: physical activity; MVPA: moderate-to-vigorous PA; TV: television

Only variables significantly correlated to either MVPA or screen time are presented.

* *P *< 0.05; ^# ^*P *= 0.053

## Discussion

The purpose of this study was to examine the reliability and validity of a questionnaire to measure child- and parent-reported psychosocial and environmental correlates of physical activity and screen-based behaviors among Chinese children in Hong Kong. The results suggest that the test-retest reliability was generally acceptable for individual items in both child-report and parent-report questions, as well as the emergent scales which included the self-efficacy scale, the family support scale, the peer support scale, the rules and guidance on screen-based behaviors scales, the perceived neighborhood safety scale, and the social environment scale. These scales showed evidence of convergent validity for most of the items; apart from one item in the child-reported self-efficacy scale and two items in the physical activity support scale, most items loaded onto factors within the scales. The criterion validity analysis also provided psychometric support for using the instrument to measure psychosocial and environmental correlates of physical activity among Chinese children. The results warrant further research into the development of more robust measures, especially peer influences on physical activity and self-efficacy specific to screen-based behaviors.

Since there is no existing assessment scheme specifically developed for Chinese children, most of the items incorporated in this study were modified from published studies, where possible, in the attempt to cover most of the domains identified by ecological theory to be important. Self-efficacy has been consistently reported as a strong correlate of physical activity among children [6]. Self-efficacy measures in the current study were adapted from the existing items specifically designed to examine children's confidence in finding and creating an environment to support their physical activity [25]. Ryan and Dzewaltowski have found that these specific components of self-efficacy had the strongest associations with physical activity, compared with other types of self-efficacy, e.g. overcoming barriers to physical activity, among 11- to 13-year-old American children [25]. However, the results from EFA in the current study showed that one of the five self-efficacy items ("*I can walk to school instead of having to wait for a ride*") did not seem to be part of the construct and thus stood alone in analysis. Whether or not being an active commuter to school is an important aspect of self-efficacy for participating in physical activity among Chinese children warrants further investigation. However, validity analysis showed that there was no correlation of this specific item with physical activity (data not shown), whereas, a positive association was found between the self-efficacy scale generated by taking the average of the other four items and physical activity (*r *= 0.25). It is therefore prudent to exclude this item in further studies examining self-efficacy and physical activity in Chinese children. Furthermore, as the self-efficacy scale in the current study is only relevant to children's physical activity, future research should aim to develop similar construct specific to screen-based behaviors.

It has been suggested that the support of family and peers are important correlates of physical activity among children [6,34,35]. Measures of family and peer support used in this study were adapted from the measures developed for use with Australian children [21]. The kappa statistics in the present study showed substantial test-retest reliability for most of the individual items, and were comparable to the original measures developed by Hume et al. [21]. Two of the items "*Being physically active with grandparents*" and "*Receive encouragement from a family member*" did not load onto the same constructs as the other items or had low loading and therefore was excluded. This was possibly due to relatively low report rates in these two items. The finding that the items on family and peer support reflected two different constructs was similar to what have been found in Iranian girls [18]. In the current study, the peer support scale showed lower Cronbach's alpha (0.50), which was likely due to the limited items in this construct. However, the peer support scale was associated with children's MVPA, whereas the parents' support measure was not. As peer support is closely related to physical activity in school children, especially as they approach adolescence [4,6], further research is needed to develop and test a broader range of peer support questions relating to Chinese children. The home physical activity environment consisted of newly-developed questions in the current study. Although no association with a similar construct has been found in previous studies with Caucasian children's physical activity, this factor was associated with MVPA in Hong Kong children.

Three measures of neighborhood-level influences on children's physical activity were adapted in this study from existing instruments (i.e., availability of sports facilities, safety concerns, and the social environment). Similar constructs have been extensively examined among Caucasian youth in literature [4,6,16,17,36-41]. In the current study, the Chinese children were able to consistently report on the available sports facilities in their neighborhood. The finding that the safety scale and the social environment scale showed good internal consistency was consistent with previous studies [21,28] and emphasizes that a range of questions should be included in order to assess the different aspects of the local environment.

Unlike the correlates of physical activity, studies focusing on correlates of sedentary behavior, in particular, screen-time among children, have been limited [6]. In this study, the test-retest ICC values of the individual items assessing parental rules and guidance in relation to children's screen-time were comparable to the original measures developed by Salmon et al. [15], and the overall rules scale and guidance scale showed substantial reliability. However, unlike the study in Australian children showing that parents' rules prohibiting TV viewing during meal times was negatively related to children watching TV > 2 hr/day [15], the rules and guidance scales were not associated with screen-time among Chinese children. It has been shown in a recent review [6] that two of three studies examining correlates of screen-based behaviors found associations between parent TV time and their child's sedentary behaviors, A similar positive association was also observed in the current study between parental modeling of TV and children's screen-time. It was also interesting to note that family support for physical activity was negatively associated with children's screen time in the current study. These results support the notion that sedentary behavior and physical activity may have their own determinants [6]. Identifying more measures is necessary in future research to examine the correlates of sedentary behaviors among Chinese children.

### Strengths and Limitations

This study has several implications for practice. The questionnaire developed in this study provides a useful tool for health professionals, researchers, and school policy makers to examine the multiple correlates of physical activity and screen-time among Chinese children. Based on a social ecological framework, the questionnaire could provide an overall understanding of psychosocial and environmental factors that may shape and change children's physical activity behavior. Further, this questionnaire can provide information on the relative contributions that these factors make to children's overall physical activity levels; thus informing the most to least important factors to target in physical activity promotion initiatives. This questionnaire can also be used in other Asian countries which share similar cultural and environmental characteristics and manifest similar health problems among their inactive youth population.

The results of this study also have implications for tailoring the interventions to increasing lifestyle physical activity among Chinese children. Although correlation analyses were not the focus of this study, validity results showed some evidence of associations with physical activity. For instance, self-efficacy continues to be a significant correlate of children's physical activity as consistently shown in previous studies [9]. Children who reported more support from their peers seemed to be more physically active; although as this was a cross-sectional association, the reverse association could also be true. Family support for physical activity might also be important for children to reduce their screen time. These findings indicate that multiple factors, including individual, social, and environmental aspects, should be considered in order to develop the appropriate behavioral change strategies among Chinese children.

There are several limitations with this study. Firstly, a small number of emergent scales, e.g. peer support, had relatively low Cronbach's alphas. There were only two items relevant to peer influences on physical activity, which inevitably limits the ability to adequately measure this construct. Future research should aim to develop more robust measures of this construct. The other research challenge, which is similar to previous studies, relates to the definition of a neighborhood [42]. This was apparent among the children when asked about access to sports facilities in their community. In the present study, children were guided to think about a "neighborhood" as being the area within a 20-minute walk or drive from their home in order to help them recall the geographic area that they are familiar with. However, it may be that the recreational destinations that children usually go to for physical activity may not necessarily be within the defined area [43]. Future research exploring the neighborhood environmental correlates of physical activity among Chinese children should consider using objective measures in addition to self-report measures.

## Conclusions

In summary, the results provide psychometric support for using a questionnaire to measure child-report and parent-reported psychosocial and environmental correlates of physical activity among Chinese children in Hong Kong. It seems that the measures of correlates of physical activity that have mainly been developed for application among Caucasian children could be transferable to their counterparts in an Eastern country after some revisions. However, more robust development and investigation of the measurement properties of the correlates of physical activity and screen-based behaviors is required for Hong Kong children.

## Competing interests

The authors declare that they have no competing interests.

## Authors' contributions

YJH conducted the literature review, drafted questionnaire, collected and analyzed data, and drafted the manuscript; SHW participated in the development of the questionnaire and the design of the study, and edited the manuscript; JS participated in revision of the questionnaire; both JS and SSH provided substantive feedback on the manuscript. All authors read and approved the final manuscript.

## Supplementary Material

Additional file 1 **Description of the psychosocial and environmental measures in the questionnaire**. The file describes all the measures and abbreviated questions developed in the questionnaire. Click here for file

Additional file 2 **Factor pattern for the emergent scales**. This file provides the results from EFAs, including factor loadings, communality coefficients and Kaiser-Meyer-Olkin values. Click here for file
