# Supplementary material for: Reliability and validity of psychosocial and environmental correlates measures of physical activity and screen-based behaviors among Chinese children in Hong Kong
Source: Int J Behav Nutr Phys Act. 2011 Mar 8;8:16. doi: 10.1186/1479-5868-8-16 (PMC3065395; doi:10.1186/1479-5868-8-16)
Supplement: Additional file 1 — Description of the psychosocial and environmental measures in the questionnaire. The file describes all the measures and abbreviated questions developed in the questionnaire. [file 1479-5868-8-16-S1.DOC]

## Additional File 1: Description of the psychosocial and environmental measures in the questionnaire

| **Name of variables** | **No. of items** | **Descriptions of abbreviated questions** | | | **Report status** |
| --- | --- | --- | --- | --- | --- |
| ***Psychosocial*** |  |  | | |  |
| Self-efficacy* | 5 | Agreement with: *I am confident that I can*…  Find people to be physically active with; find a park near my house or other place to be active; walk to school instead of having to wait for a ride; ask parents to make room for PA in my house; ask parents to buy sports wear or a racket  (‘1’, totally disagree to ‘5’ totally agree) | | | C |
| Home PA environment | 2 | *Do you have the followings in the home?*  Sports facilities; sports clothes  (‘1’, yes or ‘0’ no) | | | C |
| Family and peer support for PA* | 8 | *Do the following people usually be physically active with you?*  whole family; father; mother; grandparents; siblings; friends  *Do a friend or family member usually offers you encouragement to be physically active?*  from a family member; from friends  (‘1’, yes or ‘0’ no) | | | C |
| Perceived parental enjoyment of screen-based behaviors together | 3 | Agreement with: my parents enjoy watching TV; using the computer; playing electronic games together with me  (‘1’, totally disagree to ‘5’ totally agree) | | | C |
| Parental role modeling | 3 | Estimated time in watching TV (<2 hr⋅d^-1^, 2-4 hr⋅d^-1^ and ≥4 hr⋅d^-1^); using computer (<30 min⋅d^-1^ or ≥30 min⋅d^-1^); participating in MVPA in a typical week (<30 min⋅d^-1^, 30-60 min⋅d^-1^ or >60 min⋅d^-1^) | | | P |
| Rules and guidance on children’s screen-based behaviors* | 6 | Agreement with: control the time my child spend in TV, computer & Internet use; no TV is allowed during meal time; not allow TV until homework done; provide guidance when my child play computer games, watch TV and use Internet  (‘1’, totally disagree to ‘5’ totally agree) | | | P |
| Sedentary opportunities in the home | 5 | *Do you have the followings in the home?*  TV; video/DVD player; electronic games; computer; Internet access; TV set in the child’s bedroom  (‘1’, yes or ‘0’ no) | | | P |
| ***Environmental*** | |  |  |  | |
| Perceived neighborhood safety* | 5 | Agreement with: it is easy to walk around; it is safe area to walk; the roads are safe; feel safe crossing the roads; worried about strangers (reversed)  (‘1’, totally disagree to ‘5’ totally agree) | | | C |
| Social environment in the neighborhood* | 4 | Agreement with: I have many friends in my area; there are lots of children around to play with; I know many people in my area; I know many of my neighbours quite well  (‘1’, totally disagree to ‘5’ totally agree) | | | C |
| Sports facilities in the neighborhood | 5 | *Do you have the followings in the area where you live?*  Park; swimming pool; bike track; outdoor sport fields; indoors gymnastics  (‘1’, yes or ‘0’ no) | | | C |

C: child-reported; P: parent-reported; PA: physical activity; MVPA: moderate-to-vigorous PA; TV: television

*The emergent scales in the questionnaire, the results from exploratory factor analyses (EFAs) are provided for these scales in Additional File 2.
