# Supplementary material for: Reliability and validity of psychosocial and environmental correlates measures of physical activity and screen-based behaviors among Chinese children in Hong Kong
Source: Int J Behav Nutr Phys Act. 2011 Mar 8;8:16. doi: 10.1186/1479-5868-8-16 (PMC3065395; doi:10.1186/1479-5868-8-16)
Supplement: Additional file 2 — Factor pattern for the emergent scales. This file provides the results from EFAs, including factor loadings, communality coefficients and Kaiser-Meyer-Olkin values. [file 1479-5868-8-16-S2.DOC]

## Additional File 2: Factor pattern for the emergent scales

|  | **Factor loadings** | ***h*^2^** | **Kaiser-Meyer-Olkin value** |
| --- | --- | --- | --- |
| **Self-efficacy** |  |  | 0.686 |
| Find people to be physically active with | 0.604 | 0.486 |  |
| Find a park near my house or other place to be active | 0.657 | 0.472 |  |
| Walk to school instead of wait for a ride | *0.174* | *0.083* |  |
| Ask parents to make room for PA in my house | 0.669 | 0.453 |  |
| Ask parents to buy sports wear or a racket | 0.815 | 0.944 |  |
| **Perceived neighborhood safety** |  |  | 0.711 |
| It’s easy to walk around | 0.634 | 0.402 |  |
| It's a safe area to walk | 0.746 | 0.556 |  |
| The roads are safe | 0.832 | 0.693 |  |
| Feel safe crossing the road | 0.687 | 0.472 |  |
| Worried about strangers (reversed) | 0.361 | 0.130 |  |
| **Social environment in the neighborhood** |  |  | 0.650 |
| I have many friends in my area | 0.721 | 0.519 |  |
| There are lots of children around to play with | 0.785 | 0.616 |  |
| I know many people in my area | 0.749 | 0.561 |  |
| I know many of my neighbors quite well | 0.733 | 0.538 |  |

|  | **Factor 1 loadings** | **Factor 2 loadings** | ***h*^2^** | **Kaiser-Meyer-Olkin value** |
| --- | --- | --- | --- | --- |
| **Rules and guidance on children’s screen-based behaviors** | **Guidance** | **Rules** |  | 0.728 |
| Control time in TV, computer & Internet use | *-0.091* | 0.554 | 0.278 |  |
| No TV during meal time | *0.073* | 0.617 | 0.419 |  |
| Not allow TV until homework done | *0.229* | 0.320 | 0.208 |  |
| Guidance during computer games | 0.782 | *-0.029* | 0.596 |  |
| Guidance during TV watching | 0.908 | *-0.073* | 0.781 |  |
| Guidance during Internet use | 0.708 | *0.105* | 0.567 |  |
|  | *Factor correlation=0.369 | | | |
| **Family and peer support for PA** | **Family support** | **Peer support** |  | 0.717 |
| Whole family was physically active together | 0.838 | *-0.154* | 0.637 |  |
| Physically active with father | 0.537 | *0.096* | 0.333 |  |
| Physically active with mother | 0.604 | *-0.037* | 0.350 |  |
| Physically active with grandparents | *0.201* | *0.175* | 0.095 |  |
| Physically active with siblings | 0.349 | *-0.017* | 0.118 |  |
| Physically active with friends | *-0.045* | 0.425 | 0.169 |  |
| Receive encouragement from a family member | *0.224* | *0.171* | 0.106 |  |
| Receive encouragement from friends | *0.015* | 0.578 | 0.340 |  |
|  | *Factor correlation=0.341 | | | |

Principal axis factoring

*When more than one factor was determined, direct oblimin rotation was conducted as the two factors were correlated.

*h*^2^: communality coefficient; PA: physical activity; TV: television

Italic: item having low factor loading <0.30
